# Functional Genomics of Allergen Gene Families in Fruits

**DOI:** 10.3390/nu1020119

**Published:** 2009-10-28

**Authors:** Fatemeh Maghuly, Gorji Marzban, Margit Laimer

**Affiliations:** Plant Biotechnology Unit, IAM, Department of Biotechnology, VIBT BOKU, 1190 Vienna, Austria; Email: fatemeh.maghuly@boku.ac.at (F.M.); gorji.marzban@boku.ac.at (G.M.)

**Keywords:** temperate fruits, small fruits, pathogenesis-related proteins, Southern Blot, RT-PCR, ELISA, Immuno Tissue Printing, Western Blot, 2D-electrophoresis, *Agrobacterium* mediated transformation, RNAi approach

## Abstract

Fruit consumption is encouraged for health reasons; however, fruits may harbour a series of allergenic proteins that may cause discomfort or even represent serious threats to certain individuals. Thus, the identification and characterization of allergens in fruits requires novel approaches involving genomic and proteomic tools. Since avoidance of fruits also negatively affects the quality of patients’ lives, biotechnological interventions are ongoing to produce low allergenic fruits by down regulating specific genes. In this respect, the control of proteins associated with allergenicity could be achieved by fine tuning the spatial and temporal expression of the relevant genes.

## 1. Introduction

In general, fruit consumption is encouraged for health reasons. However, fruits may harbour a series of allergens, responsible for hypersensitivity to food to a different extent in sensitized individuals. Plant-derived foods can trigger allergic reactions with symptoms ranging from mild and localized oral allergic syndrome (OAS) to severe life-threatening anaphylactic shock and death [1]. Clinical studies performed in Europe suggest that 2–4 % of the adult and 6–8 % of the children population are affected by food allergies, which is comparable to 3.5 to 4 % of the US adult population suffering from IgE mediated food allergy [2,3,4,5,6,7,8,9]. Physicochemical properties of fruit allergens and their resistance to proteolysis in the digestive tract seem to be a key factor to induce direct sensitizations through the oral route, as well as systemic reactions upon ingestion [10,11]. Therefore a valid food risk assessment requires detailed knowledge about the allergens present in fruits and their properties. A few fruit proteins which are unaffected by gastric digestion are absorbed in the intestine and can induce sensitization and allergic symptoms as well [12]. Such proteins are called “complete or real food allergens” or class I food allergens, due to their ability to sensitize and to elicit allergic reactions. Some proteins, however, are known to provoke symptoms, but usually do not sensitize. These allergens are defined as “incomplete or cross-reactive allergens” or class II food allergens, provoking allergic reactions only in sensitized individuals due to cross-reactivity to the corresponding sensitizer [13,14,15,16]. In fact, most allergic patients react to more than one single allergen, the reasons for this allergenic multi-reactivity being either multiple sensitization events and/or cross-reactivity [17,18] or structural similarities. Cross-reactivity requires more than 70 % sequence identity [13], which exists in conserved sequences resulting in nearly identical epitopes in allergens of related and unrelated plant taxa [19]. 

A variety of allergens from different fruits were identified based on experimental immunology and molecular biology, i.e., by sequencing, leading to gene and protein identification. Among the different fruit allergens, the pathogenesis-related (PR) proteins, classified into 17 families based on sequence, diverse structure, function and biological activity [20], are produced in response to different biotic and abiotic stresses (Table 1). Members of several families were shown to play a role in plant defence and allergenicity [21,22]: a) the PR-2 proteins (β-1,3-glucanases) and b–c) the PR-3 and PR-4 proteins (chitinases), enzymes that hydrolyse β-1,3-glucans and chitin, respectively [23,24]; d) the PR-5 proteins [thaumatin-like proteins (TLP) and osmotins], which display a clear antifungal and/or membrane-permeabilizing activity [25]; e) the biological function of the PR-10 proteins is still unknown [26]; f) the family of PR-14 proteins are characterized as non-specific lipid transfer proteins (nsLTPs) and are named according to their ability to transfer lipids between membranes and to bind fatty acids [27].

Since PR-10 proteins are degraded during the passage through the gastrointestinal tract, they cannot directly induce sensitization. Some authors claim that PR-10 proteins cause allergy only in patients previously sensitized by common epitopes of Bet v 1, the birch major pollen allergen [16]. However, when conformational epitopes of Bet v 1 were stabilized, hypersensitivity could be induced via oral route in mice [28]. Contrarily, the PR-14 proteins are very stable and responsible for true food allergies upon consumption of rosaceous fruits, i.e. apple peach, cherry, apricot and strawberry, particularly in the Mediterranean area [10,29,30,31]. Due to a high refolding and renaturation capacities, PR-5 proteins are the second group of stable allergens [32]. TLPs and their clinical relevance are currently under investigation; however there are poor data on demographic differences [10]. Profilins are highly conserved 12–15 kDa proteins, easily degraded by proteases, and a major cause for cross reactivity between pollen and plant-derived food [33]. A systematic study about the clinical relevance of profilins showed more importance for Southern European populations than for Central and Northern Europe [10]. 

**Table 1:** Families of PR proteins according to [20].

Families	Type member	Properties	Gene symbol
PR-1*	Tobacco PR-1a	Unknown	*Ypr1*
PR-2*	Tobacco PR-2	β-1,3-glucanase	*Ypr2, [Gns2 (‘Glb’)]*
PR-3*	Tobacco P, Q	Chitinase type I,II, IV,V,VI,VII	*Ypr3, Chia*
**PR-4**	Tobacco ‘R’	Chitinase type I,II	*Ypr4, Chid*
PR-5*	Tobacco S	Thaumatin-like	*Ypr5*
**PR-6**	Tomato Inhibitor I	Proteinase-inhibitor	*Ypr6, Pis (´Pin´)*
**PR-7**	Tomato P_69_	Endoproteinase	*Ypr7*
PR-8*	Cucumber chitinase	Chitinase type III	*Ypr8, Chib*
**PR-9**	Tobacco “lignin-forming peroxidise”	Peroxidase	*Ypr9, Prx*
PR-10*	Parsley “PR1”	Ribonuclease-like	*Y pr10*
**PR-11**	Tobacco “class V” chitinase	Chitinase, type I	*Ypr11, Chic*
**PR-12**	Radish Rs-AFP3	Defensin	*Ypr12*
**PR-13**	Arabidopsis THI2.1	Thionin	*Ypr13, Thi*
PR-14*	Barley LTP4	Lipid-transfer protein	*Ypr14, Ltp*
**PR-15**	Barley OxOa (germin)	Oxalate oxidase	*Ypr15*
**PR-16**	Barley OxOLP	Oxalate oxidase-like	*Yrp16*
**PR-17**	Tobacco PRp27	Unknown	*Yrp17*

* PRPs with allergenic potential

Fruit from some *Rosaceae* are reported to cause allergic reactions in certain individuals. Since apple is the most frequently produced and consumed fruit worldwide due to its well known health benefits and high content of vitamins, polyphenols and fibre, it was considered as a *Rosaceae* model plant [34]. Initially, four major allergens were reported in apple: 1) Mal d 1 (PRP-10), homologous to birch pollen Bet v 1 with a molecular mass of 17.5 kD; 2) Mal d 2 (PRP-5), a thaumatin-like protein (TLP), with a molecular mass of 23 kD; 3) Mal d 3 (PRP-14) a lipid transfer protein (LTP) with a molecular mass of 9 kD and 4) Mal d 4 (proﬁlin), homologous to birch pollen Bet v 2 with a molecular mass of 14 kD [8,35,36]. 

Birch pollen allergy is well-known to be commonly associated with secondary food hypersensitivity, including tree nuts, carrot (*Daucus carota*), celery (*Apium graveolens*), kiwi (*Actinidia chinensis*), and soybean (*Glycine max*)Osterballe 2, but also fruits of the Rosaceous family like stone fruits (*Prunus sp*.), apple (*Malus domestica*), strawberry (*Fragaria* x *ananassa*) and raspberry (*Rubus idaeus*) [31,37,38]. Recently even evidence for a strikingly close relationship between birch pollen allergy and fig (*Ficus carica*) intolerance was provided [39]. Although primary sensitization is assumed currently to occur through pollen exposure, there are only few investigations of pollen from woody crop species. Investigation of fruit pollen allergen content suggested an alternative route of sensitization to major fruit allergens like PR-10 and PR-14 proteins direct inhalation. Both allergen families were detected in pollen of various Rosaceous fruit trees at both the protein and gene level [40]. 

## 2. Genomic Approach

**Figure 1:**
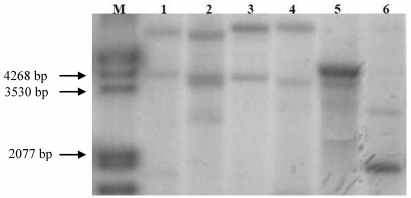
Genomic DNA digested with *EcoR*I, hybridized with Mal d 3-DIG labeled probe, M) molecular weight marker III, 1) apple, 2) plum, 3) cherry, 4) strawberry, 5) raspberry, 6) blackberry.

The large-scale genomic sequencing in *Rosaceae* revealed a high similarity among crop species in this family and contributed to the discovery of novel putative fruit allergens [41]. Based on their homology to PR-10 and PR-14 proteins in apple, i.e., Mal d 1 and Mal d 3, the genomic organization of PR-10 and PR-14 proteins in blackberry, cherry, plum, raspberry and strawberry was analyzed by Southern blotting with known apple sequences as probes  (Figure 1), which might be indicative for the expected variability and presence of isoforms. Also, Southern blot analyses indicated that LTP are encoded by at least two genes [42]. A BLAST pairwise alignment based on PR-10 and PR-14 genomic sequences between of raspberry (*Rubus ideaeus* L.), Rub i 1, Rub i 3 and the NCBI database sequences showed different subfamilies of both genes and the relationship between homologous sequences (Appendix Figures Ι (a-b)). Conversion of DNA sequences of Rub i 1 (Acc. no. DQ660361) and Rub i 3 (Acc. No. DQ660360) into amino acid sequences using primers designed on the basis of high sequence homologies to Mal d 1, Mal d 3, revealed a high similarity to homologous allergens in related Rosaceous species like strawberry, cherry, apple and peach [19]. The Mal d 1 homologous protein from raspberries showed highest identity to cherry allergen Pru av 1 (79.0%) and strawberry Fra a 1 (77.0%) followed by peach Pru p 1, apple Mal d 1 and apricot Pru ar 1. Sequence alignment of Rub i 3 to PR-14 proteins from other *Rosaceae* fruit showed also highest identity with the allergens Fra a 3 (identity 87%) and Mal d 3 (identity 74%) [19]. This protein shares also 8 cysteine-residues, which are crucial for the conformational stability of this allergen, with other LTPs like Pru av 3, Pru p 3 and Pru ar 3 from apricot, cherry and peach respectively [43].

Although food allergens belong to a limited number of protein families, each protein family could encoded by multiple genes [44]. Considering the important role played by LTP in mediating plant signal transduction, the expression of *Ltp* gene was investigated in *Prunus incisa × serrula* (PIS) under various abiotic stress conditions (low and high temperatures, salicylic acid and wounding stress, harvested after 0, 0.5, 1, 2, 4, 10, 24 and 72 h). Results obtained suggest that *Ltps* are differentially regulated in response to different stresses in PIS plants, and additionally show a tissue-speciﬁc expression, hinting at a potential role of different isoforms [42]. PR-10 mRNAs in apple are encoded by a multigene family, transcribed in a similar size; however, a considerable diversity in their deduced amino acid sequence was demonstrated [25,45,46,47]. Using isoform-specific primers the expression proﬁle of the four major isoform clusters were studied in apple fruits and a clear pattern concerning the prevalence of isoforms was obtained and showed variable expression ratios in fruit and vegetative tissues [48]. Isolation and characterization the promoter of a Mal d 1 (PR-10 protein in apple) from the genomic clone was shown to be stress and pathogen-inducible [25] and different isoforms could be up-regulated in young leaves upon stress attack. However, its biological functions, as that of many PR-10 proteins, are still unknown. In order to identify proteins putatively interacted with Mal d 1 an expression library was screened using a two- hybrid system approach which a PR-10 associated protein (MdAP) was identified [48]. 

## 3. Development of Patient Independent Detection Tools for Characterization of Fruit Allergen

It is important to note, that the protein extraction from fruit tissue represents the major bottleneck in allergen analyses. A conditional factor for electrophoretical separation is to keep the proteins in a solubilised state. An ideal protein extraction protocol captures all proteins in a given sample, whilst removing non-protein or interfering impurities, requiring different approaches. Complex buffer according to [49], one and multiple step precipitations using phenol or/and TCA (trichloracetic acid) have been often used for the extraction of plant proteins and allergens [49,50,51]. Since the comparison of previous procedures showed completely different bands and spot arrangements in SDS-PAGE and 2DE gels, for each plant tissue and organ an specific extraction procedure must be established [51]. 

**Figure 2:**
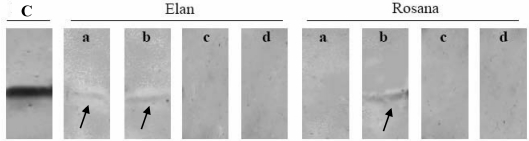
Influence of TCA-precipitation and sample preparation procedures on the detection of strawberry LTP (Fra a 3) from two different cultivars (Elan and Rosana) using four different sample preparation procedures: a) 1% SDS + 50 mM DTT, b) 7 M urea + 50 mM DTT, c) 1% SDS, d) 7 M Urea; C: native apple LTP as positive control.

The detection of LTPs required specific optimizations of the extraction procedure from apricot [50] and from strawberry, *Fragaria* x *ananassa*. Native Fra a 3, LTP from strawberries, could not be isolated from strawberry tissues, however, a recombinant Fra a 3 was cloned and expressed in the yeast *Pichia pastoris* for immuno-blot-inhibition tests [33]. Different strategies to detect LTP from strawberries showed that three factors interfere with protein extraction: a) the type of cultivar and plant specific matrices, b) the procedure of protein extraction and c) the procedure of sample preparation. TCA-precipitation and addition of a reducing agent allowed the detection of LTP from cultivar Elan, grown in the greenhouse, however after using the same extraction and sample preparation procedure for cultivar Rosana, the LTPs could only be detected in the presence of urea and DTT (Figure 2). The experiment revealed the importance and the influence of cultivar specific matrices on the efficiency of allergen detection. 

**Figure 3:**
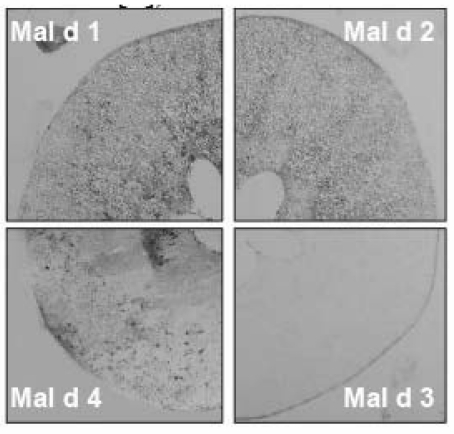
Immuno-tissue prints allowed the localization of the four major apple allergens within the apple tissue (adapted from [34]).

To localize the four main allergens in apple tissues the immuno-tissue print was developed [34]. Mal d 3 is the only allergen exclusively localized in the peel. The other allergenic proteins are equally distributed in peel and pulp (Figure 3). A sandwich ELISA using monoclonal antibody directed to Mal d 1 delivered data about the intra and inter apple variability in different cultivars (data not shown). The measurements showed significant differences (up to 2-fold) among apples of the same cultivar and (7-fold) within one single Jonagold apple [34]. The inﬂuence of seasonal, regional and cultivation-related factors on the level of allergen carried out over a period of three consecutive years in two different locations showed large fluctuations and requires several years of data gathering and higher sample numbers to allow a conclusive interpretation [34].

Using polyclonal antibodies the presence of Mal d 1 and Mal d 3 homologues of the major apple allergen Mal d 1 and Mal d 3 were identified in variable amounts in pollen extracts of apple, apricot, cherry, peach, strawberry, raspberry, blackberry, quince, medlar, pear, sour cherry and rose (*Rosaceae*), elder (*Caprifoliaceae*), blueberry (*Ericaceae*), orange (*Rutaceae*), carrot (*Apiaceae*), grape (*Vitaceae*), cornel cherry (*Cornaceae*), mulberry (*Moraceae*), olive (*Oleaceae*), pomegranate (*Lythraceae*) (data not shown).

SDS-PAGE and two-dimensional electrophoresis (2-DE) were successfully established for allergen mapping of apples [35]. The application of 2-DE technique led further to the characterization of putative raspberry allergens like Rub i 1 (a Bet v 1 homologous protein), Rub i 3 (a LTP), class III acidic chitinase and cyclophilin [19]. The comparison of amino acid sequences of these two proteins with other rosaceous fruit sequences showed significant identities at the amino acid sequence level, indicating a strong potential cross reactivity [19]. 

## 4. Detection Tools for Characterization of Fruit Allergens Involving Patient Sera

**Figure 4:**
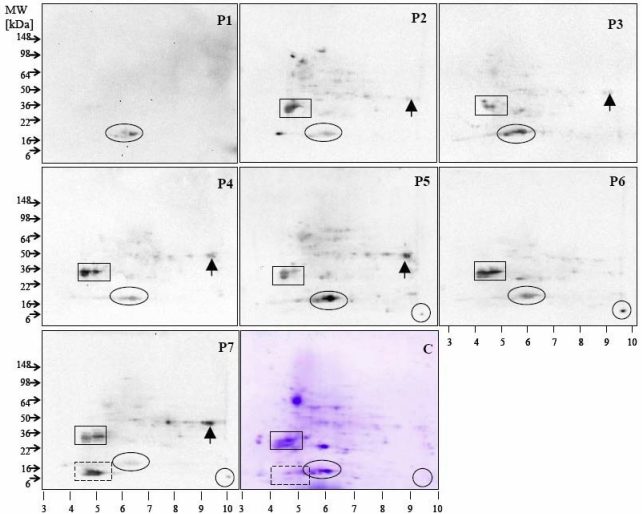
Identification of allergenic proteins using sera of seven patients (P1–P7). The allergens are highlighted using an ellipse showing Mal d 1, a rectangle for non-reduced Mal d 2, a circle for Mal d 3 and a dotted rectangle for Mal d 4, arrow correspond to the newly identified glyceraldehyde-3-phosphate dehydrogenase by mass spectrometric analysis and *de novo* peptide sequencing, showing reactivity to 71% of the tested sera, which previously identified as a minor allergen in wheat [50]. The Coomassie-stained 2-DE gel of the apple extract is shown in (C).

To allow a conclusive classification as allergen, the detection data obtained using mouse monoclonal or rabbit polyclonal sera require a confirmation with sera from allergic individuals [52]. SDS-PAGE and Western blotting techniques in one- and two-dimensional electrophoreses are used due to their flexibility and ease of use [34,35]. For the identification of individual protein spots, mass spectrometry and *de novo* peptide sequencing are routinely used. Both the use of 2-DE and mass spectrometry provides a component resolved map of individual patient-related sensitisation patterns and offers as unique opportunity a link to complementary computational and bioinformatics tools (Figure 4). 

## 5. Strategies towards Low-Allergenic Fruits

Conventional breeding approaches are currently not feasible for allergen reduction in fruits, due to the long term undertaking, the incomplete understanding of the genetic background and the limited genetic resources. Indeed the degree of susceptibility towards biotic and abiotic stress factors varies with cultivars, climatic conditions and possibly with other factors that affect the synthesis and accumulation of defence proteins and protective mechanisms. 

Over the past few decades, the possibilities for improvement of germplasm have been broadened by extensive gene mapping and identification, whole-genome sequencing of model plants and crops and the use of gene transfer technologies [16,53]. Genetic engineering provides advantages beyond classical breeding, not only by increasing the scope of gene and the types of mutations that can be introduced, but also due to the potential control the spatial and temporal expression patterns of genes of interest. 

In many crops, the tissue that is mainly consumed as food, e.g., fruits, is distinct from the tissues determining plant growth and productivity, i.e., roots and shoots. In many cases, however, genes controlling specific traits do not operate in a tissue-specific manner, but function in many different plant organs. Therefore, a modification of a gene improving fruit quality might harbour unexpected effects on growth and development of other plant organs.

RNA-induced gene silencing is an intriguing alternative approach to circumvent these limitations, also termed post-transcriptional gene silencing (PTGS) in plants [54]. The essence of RNA-induced gene-silencing is the delivery of double-stranded RNA (dsRNA) into an organism, or a cell, to induce a sequence-specific RNA degradation mechanism that effectively silences a targeted gene. Various biotechnological interventions are being applied to remove or to reduce plant-derived proteins that can provoke allergic reaction in human. *Agrobacterium tumefaciens* mediated transformation is currently applied at the PBU, BOKU, Vienna, to down-regulate the Mal d 1 and Mal d 3 homologues in blueberries (*Vaccinium corymbosum*). IgE reactivity to blueberry extract was determined using Western blotting and patient sera [31]. Post-transcriptional gene silencing (PTGS) was used to eliminate the allergen expression in rice and soybean which led in sequence-specific mRNA degradation and prevention of the gene translation [55]. In rice the 14–16 kDa allergen was down-regulated, but none of the transgenic plants was allergen-free. In soybeans, the application of this strategy resulted in a complete knockdown of soybean allergen was obtained. The genetic elimination was maintained over three generation and there were no significant morphological or reproductive degeneration compared to wild type. To minimize the hypersensitivity to apples, a RNAi approach was recently applied to down-regulate the major apple allergen Mal d 1 [56]. The transgenic lines showed up to 10-fold reduction in Mal d 1 leaf expression without any phenotypic differences compared with wild type. The same strategy was used to down regulate successfully two tomato allergens, the Lyc e 1 (profilin) and Lyc e 3 (LTP). However, the phenotypes were significantly different from wild type [57,58]. 

Food production of high quality standards and environmental safeguarding concerns set new requirements for a modern plant production system. The apparent conflicting situation, i.e. to produce fruits without compromising the natural defences of the plants and the quality of fruits - a approach should be chosen, where it is possible to rely on a delicate balance between the traits expressed in the fruit tissues during different developmental stages. The control of proteins associated with allergenicity will be achieved by a fine tuned control of the spatial and temporal expression of genes coding for PR proteins, without compromising the plant response to biotic and abiotic stresses.

## Figures and Tables

**Figure Ι:**
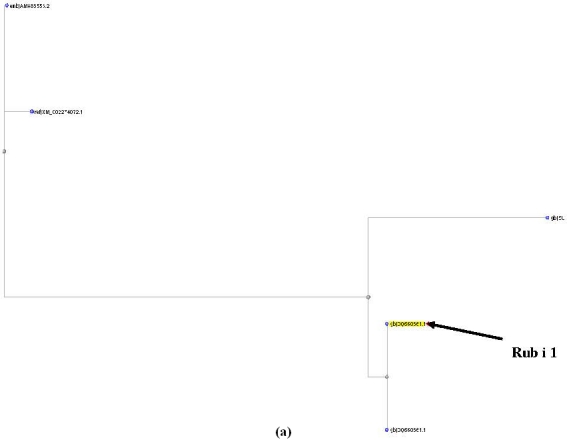

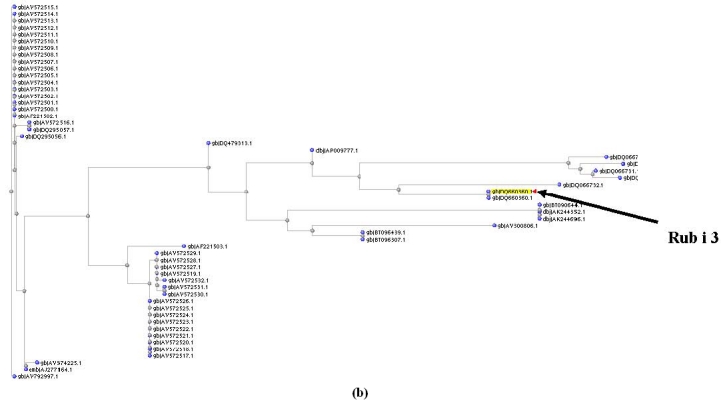
A BLAST pairwise alignment based on genomic sequences between a) Rub i 1; b) Rub i 3 and the NCBI database (GenBank accession number)
